# Tobacco retail availability and smoking behaviours among patients seeking treatment at a nicotine dependence treatment clinic

**DOI:** 10.1186/1617-9625-12-19

**Published:** 2014-12-02

**Authors:** Michael Chaiton, Graham Mecredy, Jürgen Rehm, Andriy V Samokhvalov

**Affiliations:** Social and Epidemiological Research (SER) Department, Centre for Addiction and Mental Health (CAMH), Toronto, Canada; Dalla Lana School of Public Health, UofT, T523, 33 Russell St., M5S 2S1 Toronto, Ontario Canada; Institute of Medical Sciences, University of Toronto (UofT), Toronto, Canada; Dept. of Psychiatry, Faculty of Medicine, UofT, Toronto, Canada; PAHO/WHO Collaborating Centre for Mental Health & Addiction, Toronto, Canada; Epidemiological Research Unit, Technische Universität Dresden, Klinische Psychologie & Psychotherapie, Dresden, Germany; Addictions Program, Centre for Addiction and Mental Health (CAMH), Toronto, Canada

**Keywords:** Tobacco, Smoking cessation, Environment, Spatial

## Abstract

**Background:**

Availability of tobacco may be associated with increased smoking. Little is known about how proximity to a retail outlet is associated with smoking behaviours among smokers seeking treatment.

**Methods:**

A cross sectional study was conducted using chart data was extracted for 734 new clients of a nicotine dependence clinic in Toronto, Canada who visited during the period April 2008 to June 2010. Using a tobacco retail licensing list, clients were coded as to whether there were 0, 1, or more than 1 retail outlet located 250 m from their postal code address. Conditional fixed effects regression analyses were used to assess the association between proximity and quit status, number of previous quit attempts, number of cigarettes per day, and time to first cigarette, controlling for demographic characteristics and neighbourhood.

**Results:**

72% of patients lived within 250 m of a retail outlet. Those who had more than one outlet with 250 m of their address were less likely to be abstinent at the initial assessment (OR = 0.45; 95% CI: 0.23, 0.87; p = 0.014) and less likely to have a longer time to first cigarette (OR = 0.60; 95% CI: 0.45, 0.79), both before and after adjustment for covariates. Smokers who had at least one outlet within 250 m of their address smoked 3.4 cigarettes more per day than smokers without an outlet after controlling for neighbourhood and covariates. There was no significant association between proximity and lifetime number of quit attempts.

**Conclusions:**

Proximity to a tobacco retail outlet was associated with smoking behaviours among a heavily addicted, treatment seeking population. Environmental factors may have a substantial impact on the ability of smokers to quit smoking.

## Background

Despite the enormous health burden, tobacco is still widely available for sale with few restrictions or requirements [1]. Widespread availability allows for reduced costs in terms of time and effort to obtain tobacco [2]. Further, availability may be associated with more point of sale marketing of tobacco products [including price promotions] [3]. For former smokers, receiving cues to smoke in places where they regularly shop may also contribute to high levels of recidivism [4].

There is, however, limited information on the observed effect of tobacco retail availability on individual level smoking behaviours. A Finnish study found that proximity (distance to the closest retailer) but not density (number of retailers within a specific area) was related to abstinence among male smokers [5]. Reitzel et al. [6] found that smokers in a smoking cessation trial who lived within 250 m and 500 m of a convenience store were less likely to quit smoking than other smokers who lived further away from a convenience store [6]. On the other hand, studies from Scotland [7] and New Zealand [8] found no association between proximity or density and smoking cessation.

The purpose of this cross-sectional study is to examine the differences in smoking behaviour by those who live within close proximity of one or more tobacco outlets among smokers attending a specialized nicotine dependence treatment clinic in Toronto, Ontario. This unique community-based population allows the examination of the effects of tobacco retail availability among high risk smokers who have difficulty quitting in a natural, rather than a clinical trial based, setting. While a licence is required to sell tobacco, Ontario has few restrictions on the availability of smoking except for restrictions in pharmacies and establishments containing a pharmacy, public and private hospitals, psychiatric facilities, residential care facilities and by vending machine [2].

## Methods

### Data sources

A list of retail outlets was obtained from the Ontario Ministry of Health and Long-Term Care (formerly Ministry of Health Promotion and Sport) Tobacco Information System database of tobacco-selling vendors, current to 2011. Ground truthing of the retailer list was conducted in four randomly selected Forward Sortation Areas (FSA: an area defined by the first three digits of the postal code). A data collector visited all potential tobacco retailers to verify sale of tobacco within the area, including convenience stores, gas stations, grocery stores/supermarkets, discount stores, or independent bar/restaurants identified by sight and through the Yellow Pages™ online directory (http://www.yellowpages.ca). Sensitivity of the list was extremely high at 98% (57/58 of the list were selling tobacco). Specificity was 88% (57 of the 65 stores found to be selling tobacco were on the list).

Data was also obtained from the Centre for Addiction and Mental Health (CAMH) nicotine dependence clinic located in Toronto, Canada, which serves between 350–700 new and 700–1200 unique clients per year. Clients are mostly self-referred, though many of them are referred by family physicians, Smoker’s Helpline, and other CAMH programs. The clinic provides comprehensive assessment of smoking behaviours and provides clients with tailored medical and psychosocial support. For this study, three reviewers extracted data from the clinical charts of 796 new clients who attended the clinic between April 2008 and June 2010 (for further details, see Samokhvalov et al. [9]). Ethical approval for this study was provided by the Centre for Addiction and Mental Health Research Ethics Board (097/2011).

### Exposure variables

The primary exposure variable for this study was distance to the nearest retail tobacco outlet. The midpoint of the 6-digit postal codes of clinic patients who lived in urban and suburban areas were mapped using ARCGIS 10.1 (Esri). Unlike US zip codes, postal codes can identify geographic location with acceptable precision [10, 11]. Healy and Gilliand [12] found that postal codes had an average location discrepancy of 14-24 m greater than the use of geocoded address points for examining proximity to junk food locations in urban and suburban areas [12]. Circular buffers around each patient’s postal code midpoint were drawn at 250 m. Patients with zero, one, or more than one retail outlet within 250 of this midpoint were identified. The category of ‘more than one outlet’ was included to reduce potential misclassification due to postal code buffering. Valid and complete data were available for 734 patients. There were no differences in demographic characteristics between those who had data available compared to those missing (data not shown).

### Outcome variables

Through standardized clinical assessment, data were collected on number of cigarettes per day, whether or not the patient was successfully temporarily abstinent (>24 hours) at the time of initial clinical assessment, and time to first cigarette in the morning (≥20 minutes vs. < 20 minutes), number of lifetime quit attempts (≥5 vs. < 5). Patients who were abstinent at the time of the interview were seeking help in continuing their quit attempts as cessation had begun in the period between scheduling the appointment and the first visit. Time to first cigarette was reported as recalled from period of last regular smoking. Cigarettes per day were reported as current consumption at the time of interview (coded as zero for those who were attempting to abstain). Time to first cigarette and number of lifetime quit attempts were dichotomized at the 25th percentile.

### Covariates

Additional variables were collected through clinical assessment and treated as covariates for the analysis, including: age, sex, level of employment (unemployed vs. other), living with a partner (yes vs. no), categorical estimate of income (low vs. medium/high), socio-economic status (low vs. medium/high) and education level (low vs. medium/high).

### Analysis

Bivariate descriptive analyses of the study sample by retail availability (home address with zero, one or more than one outlet within 250 m) were performed using chi square tests for categorical variables and two-sample t-tests for continuous variables (comparing no outlets to one or more). A series of conditional logistic regression analyses were performed to measure the cross-sectional association between retail availability and each of the smoking behaviour outcomes (number of quit attempts, time to first cigarette, and quit status), conditional on neighbourhood as defined by the FSA. This analysis matched smokers on neighbourhood characteristics to control all common neighbourhood effects such as street density, and social economic status. A fixed effect regression model conditional on FSA was used to model cigarettes per day as a continuous variable, controlling for neighbourhood. Adjusted logistic models were also fit, controlling additionally for potential individual-level demographic covariates. All analyses were performed in SAS version 9.1 and Stata 12.

## Results

Of the 734 valid postal codes, 94% were unique to an individual. There were 84 unique FSAs with a range of 1–35 individuals per FSA (6% with only 1 person). Most addresses were within 250 m of an outlet (72%) and 57% had more than one outlet within 250 m (See Table 1). The mean age was 47 years old and 46% of the population was female. There were no significant differences in proximity to a tobacco outlet by age, income, employment status, socioeconomic status, or education; however, the demographics of the nicotine dependence clinic are significantly different than the general population, and of smokers in general [9].

**Table 1:** **Characteristics of nicotine dependence clinic patients by proximity to tobacco retail outlet, Toronto, Canada (2008–2010)**

		0 within 250 m	1 within 250 m	>1 within 250 m
Number (n)		(n = 205)	(n = 107)	(n = 422)
**Age, mean**		46.6	46.2	46.1
Number of cigarette per day, mean*		19.2	22.3	20.8
**Sex**				
	Male	43%	51%	46%
	Female	57%	49%	54%
**Income**				
	Low	72%	66%	78%
	Medium/High	28%	34%	22%
**Social economic status**				
	Low	74%	76%	81%
	Medium/High	26%	24%	19%
**Life partner (Spouse, common law)**				
	Yes	63%	72%	72%
	No	37%	28%	28%
**Education level**				
	Low (secondary and lower)	55%	53%	55%
	High (post-secondary)	45%	48%	45%
**Employed**				
	Unemployed	51%	54%	44%
	Employed, retired, student, other	49%	46%	56%
Time to first cigarette*				
	<20	59%	62%	69%
	>20	41%	38%	31%
**Number of lifetime quit attempts**				
	<5	76%	78%	75%
	>5	24%	22%	25%
Abstinent at first visit (quit)*				
	Yes	6%	3%	2%
	No	94%	97%	98%

*p < 0.05.

Those who had more than one outlet with 250 m of their address were less likely to be abstinent at the initial assessment (OR = 0.45; 95% CI: 0.23, 0.87; p = 0.014) and less likely to have a time to first cigarette greater than 20 minutes (OR = 0.60; 95% CI: 0.45, 0.79), both before and after adjustment for covariates (See Table 2).

**Table 2:** Association of residence proximity to a tobacco retail outlet (<250) and smoking behaviour outcomes (quit attempts, cigarettes per day, time to first cigarette, and quit status at interview) unadjusted and adjusted* for potential covariates among smokers attending a nicotine dependence clinic in Toronto, Canada (2008–2010)

	Quit at baseline (yes/no)		Time to first cigarette (<20 min vs. > = 20 min) ^a^		Lifetime quit attempts (<5 vs > = 5)		Cigarettes per day ^b^	
	Unadjusted	Adjusted	Unadjusted	Adjusted	Unadjusted	Adjusted	Unadjusted	Adjusted
None with 250 m	1.00	1.00	1.00	1.00	1.00	1.00	0	0
One within 250 m	0.48	0.61	0.84	0.80	0.91	0.94	3.43	3.40
	(0.12, 1.88)	(0.11, 3.34)	(0.56, 1.25)	(0.88, 1.86)	(0.60, 1.36)	(0.64, 1.40)	(0.30, 6.56)	(0.27,6.52)
> One within 250 m	0.43	0.45	0.58	0.60	1.10	1.09	2.13	1.84
	(0.21, 0.83)	(0.23, 0.87)	(0.46, 0.74)	(0.45, 0.79)	(0.72, 1.70)	(0.70, 1.72)	(-0.21, 4.47)	(-0.49, 4.19)

*Adjusted for age, sex, employment status, income level, education, socio-economic status, and living with a partner.

^a^Fixed effect logistic regression conditional odds on neighbourhood (Forward Sortation Area).

^b^Fixed effect regression parameter conditional on neighbourhood (Forward Sortation Area).

The magnitude of effect for those who had one outlet within 250 m was consistent with a dose response effect for abstinence at initial assessment and longer time to first cigarette, but there was no statistically significant effect prior to and after adjustment (see Table 2). However, smokers who had one outlet within 250 m of their address smoked 3.4 more cigarettes per day compared to smokers without an outlet whereas there was no significant effect for additional outlets.

There was no association with having only one outlet, or more than one outlet, within 250 m and lifetime quit attempts.

## Discussion

This study showed that, among heavily dependent smokers, the effect of living within 250 m of a tobacco outlet was an important correlate of smoking behaviour. It was associated with differences in cigarettes per day compared to those who did not have an outlet within 250 m. Having more than one outlet within 250 m was further related to greater likelihood of time to first cigarette being greater and being quit at the initial visit.

These findings are consistent with Reitzel et al. [5] and Halonen et al. [5] who found that smokers in closer proximity to a retail outlet were more likely to relapse. Kirchner et al. used GPS and mobile devices to assess real time exposure to points of sale of tobacco and found that those exposed to more outlets were less likely to quit successfully, highlighting that home location is a proxy for exposure and proximity to tobacco outlets [13]. Two studies that found null effects [7, 8] used store categories (supermarkets, conveniences stores, etc.) to identify locations of tobacco sales rather than relying on licensing data or other tobacco specific information. Additional longitudinal research into the effect of availability, as well as into the effect of changes in availability, on smoking behaviour are still needed.

Availability of tobacco contributes to the social perception that cigarette smoking is a normal behaviour, which may inhibit the desire to quit smoking. Their wide availability gives the impression that cigarettes are less hazardous than they are in reality or provides smoking cues to smokers trying to quit [4]. Another potential mechanism is living in closer proximity to a tobacco outlet may also affect the cost in terms of effort and time required to purchase cigarettes [2]. These nonfinancial costs have been shown to affect purchasing behaviour, as well as abstinence in the case of alcohol [14].

Studies have found that tobacco outlets are more prevalent in vulnerable neighbourhoods [15–19]. This study matched participants by neighbourhood to control for neighbourhood level effects at the FSA; however, other effects may still exist within micro-neighbourhoods. This high-risk, low socio-economic level population in this study may be more highly at risk of exposure to tobacco availability than other population groups.

One limitation of this study is that patients were not asked in this study about their consumption of contraband tobacco although anecdotally, the level of contraband tobacco use was substantial among this heavy smoking population [20, 21]. However, much contraband tobacco in Canada is sold either on or in close proximity to First Nations reserves, and the closest reserve to the clinic in our study is over 100 kilometres away. Other limitations include the use of chart extraction which was used to collect smoking history and demographic characteristics, which may be susceptible to incomplete data. The cross sectional design inherently limits the possibility of causal inference and there may have been time effects associated with changes in the retail environment over the 2 year period of the study. Finally, location information on patients was limited to their home postal code, leading to potential random misclassification, further biasing the effect towards the null.

The built environment is increasingly and consistently being shown to effect health behaviour. The availability of tobacco is an important variable that appears to affect how smokers use tobacco and their ability to quit smoking. Among this heavily dependent and unique population of smokers seeking treatment, tobacco availability was associated with adverse smoking behaviour outcomes. Policy changes to drastically reduce the widespread availability of tobacco are urgently needed.
